# The Relationship Between Epidemic Perception and Cyberbullying Behaviors of Chinese Adolescents During the COVID-19 Pandemic: Cross-Sectional Study

**DOI:** 10.2196/54066

**Published:** 2024-10-02

**Authors:** Yonggang Feng, Qihui Xue, Peng Yu, Lanxiang Peng

**Affiliations:** 1 Faculty of Education, Shandong Normal University, No.88 Wenhua East Road, Lixia District, Jinan, 250014, China, 86 531-86182177; 2 College of Ethnology and Sociology, South-Central Minzu University, Wuhan, China; 3 School of Education Science, Kashi University, Kashi, China

**Keywords:** COVID-19, epidemic perception, cyberbullying behaviors, insomnia, anxiety and depression

## Abstract

**Background:**

In response to the COVID-19 outbreak, the government initiated measures for social distancing, leading to a gradual transition of adolescents’ social interactions toward web-based platforms. Consequently, web-based behaviors, particularly cyberbullying, have become a prominent concern. Considering that adolescents experience more intense feelings, the widely increased negative emotions and strains perceived from the COVID-19 pandemic may end up engaging in cyberbullying behaviors. In addition, during the COVID-19 pandemic, adolescents experiencing insomnia and negative affect are more prone to diminished self-control, which is associated with cyberbullying behaviors.

**Objective:**

This study aims to investigate the relationship between epidemic perception and cyberbullying behaviors, while also examining the serial mediating roles of insomnia and negative affect on the relationship between epidemic perception and cyberbullying behaviors.

**Methods:**

This study presents a large-scale web-based survey conducted during the period of concentrated COVID-19 outbreaks, encompassing 20,000 Chinese adolescents. A total of 274 submitted questionnaires were discarded because of high levels of missing data or their answers were clearly fictitious or inconsistent. The final count of valid participants amounted to 19,726 (10,371 boys, age range: 12‐18 years; mean 14.80, SD 1.63 years). The Perceptions of COVID-19 Scale, Negative Affect Scale, Insomnia Scale, and Cyberbullying Behavior Scale were used to assess participants’ responses on the Questionnaire Star platform.

**Results:**

The results show that epidemic perception is positively correlated with cyberbullying behaviors (*r*=0.13; *P*<.001), insomnia (*r*=0.19; *P*<.001), and negative affect (*r*=0.25; *P*<.001). Insomnia is positively correlated with negative affect (*r*=0.44; *P*<.001) and cyberbullying behaviors (*r*=0.30; *P*<.001). Negative affect is positively correlated with cyberbullying behaviors (*r*=0.25; *P*<.001). And insomnia and negative affect play independent mediating and serial mediating roles in epidemic perception and cyberbullying behaviors.

**Conclusions:**

This study provides additional empirical evidence on the relationship between the perception of COVID-19 pandemic and cyberbullying in adolescents. In addition, the study offers recommendations for implementing interventions targeted at mitigating cyberbullying in adolescents during the COVID-19 pandemic.

## Introduction

The outbreak of COVID-19 pandemic has swept extensive infections, which has impacted the lives of people in an unprecedented way [1]. The COVID-19 global pandemic affected negatively and caused secondary damage to people’ s mental health [2,3], which may especially increase long-term adverse consequences on adolescents [4]. Currently, a key change brought by the inexorable circumstances has been a surge of pandemic-related maladaptive behaviors of adolescents [5-7], and a critical aspect that is needed to be highlighted is their web-based behaviors during the epidemic. With the implementation of social-distancing interventions, the socialization of adolescents with peers gradually transited to electronic-based platforms. There were studies that showed that the way of interaction has changed from face-to-face to web-based interaction under the impact of COVID-19 pandemic [8], and the potential for web-based behaviors–related problems to emerge or worsen during this period is high, as adolescents could use electronic devices excessively and vent their negative emotions on the web [9]. Given that, this study discusses ways in which cyberbullying behaviors could be impacted by the epidemic perception, aiming to better identify related susceptible factors and provide active approaches to promote insights into intervention suggestions.

Cyberbullying refers to an aggressive, intentional act carried out by a group or an individual, using electronic forms of contact, repeatedly and over time against a victim who cannot easily defend himself or herself [10]. It is theorized that individuals exposed to a negative stimuli sometimes use maladaptive mechanisms to cope with strain [11]. As a stressful situation factor, people’s perception of the COVID-19 pandemic might have remarkably linked with the feeling of stress [12]. During the COVID-19 pandemic, the epidemic perception reflects people’s cognitive response and evaluations for the various threats that they are or might be exposed to the epidemic [13]. Risk perception contains 2 main dimensions, namely, “dread risk” and “unknown risk” [14]. Dread risk refers to the perceived lack of control and catastrophic potential, and unknown risk reflects the unobservable of the hazard [15]. The emergence of the COVID-19 pandemic could directly arouse these 2 psychological dimensions and make people feel threatened and stressed and respond to it emotionally or behaviorally [16]. Considering that adolescents experience more intense feelings and inner influences with higher frequency and greater volatility than adults [17,18], the widely increased negative emotions and strains perceived from the epidemic may end up engaging in cyberbullying behaviors. However, at present, a part of studies has explored the relationship between psychological distress and adolescent cyberbullying, but these studies mainly emphasize the impact of the epidemic on mental health, without exploring the direct perception of the epidemic. In addition, the sample size of the study group is relatively small [19]. Thus, this study aims to investigate the influencing relation between epidemic perception and cyberbullying behaviors based on innovatively applying General Strain Theory (GST) in the specific period.

During the COVID-19 pandemic, adolescents with insomnia are more likely to struggle with greater irritability and less self-control over negative feelings, which would be related to a wide range of behavioral impairments [20,21]. Furthermore, evidences from neurobiological research revealed that the sleep disruptions of adolescents predicted decreased neurobehavioral functioning, impeding crucial behavioral and mental functions needed for adequate social interaction [22,23]. Specifically, sleep deprivation reduces activity in the prefrontal cortex (involved in executive and inhibitory processing) and its functional connectivity to the amygdala (involved in emotional responses to threat), providing a biological basis for expecting more uncontrolled, reactive aggression under threat [24,25]. As such, experiencing insomnia may have implications that extend beyond individuals to also impact their interactions with others, showing as aggressive behaviors, which could be evident on the web during the epidemic, that is, we inferred that insomnia serves as a mediator in the relationship between epidemic perception and cyberbullying behaviors on the previous research.

The psychological consequences of infectious diseases have been reported to include depressed mood, anxiety, and increased fear and stress levels [26]. It has been demonstrated that the perception of COVID-19 pandemic was associated with high rates of anxiety and depression of adolescents [27,28]. Intensive studies have revealed that negative feelings such as depression and anxiety may further predict aggressive behaviors [29,30]. On the one hand, according to the distrust schemas, depressive emotions could lead to distrust and hostile attribution of others’ intentions [31]. Thus, adolescents with depressive emotions could underestimate the acceptance and friendship of peers, which may trigger aggression [32]. Commonly, depressive symptoms are frequently accompanied by symptoms of anxiety and conduct problems stemming from anger [33]. When obsessed with depressive and anxiety emotions, adolescents might fail to express or interpret their emotional changes properly [34]. And it could be especially true under the large-scale public lockdown that forced adolescents to use electronic devices excessively and vent their negative emotions on the web. Although there have been studies to explore the impact of the epidemic on individual mental health, such as the study by Zhang et al [19] that examined the moderating effects of parent-child relationships and the negotiable fate on the relationship between psychological distress and cyberbullying, there is no study to exactly analyze the mechanism of depression and anxiety, 2 typical emotional problems. Therefore, we inferred that the impact of epidemic perception on cyberbullying behaviors of adolescents is mediated by negative affect.

Given that sleep problems are sensitively associated with the inability to use adaptive self-regulation strategies under risk situations [35], the sleep promotion could be hindered first because of the cognitive arousal and unpleasant physical reactions caused by high epidemic perception. Apart from rising stress perceived from the epidemic, the reduced physical fatigue and exposure to the sun, as well as increased use of electronic devices, directly affect sleep homeostasis [36]. Furthermore, the arousing sleep problems and insomnia could further contribute to short-term increasing in negative affect [37], which indicated that the more sleep problems individuals experienced, the higher the possibility that they might experience strong and uncontrollable negative affect during the COVID-19 pandemic. Relevant empirical researches revealed that insomnia was a risk factor for later negative affect [38].

**Figure 1.**
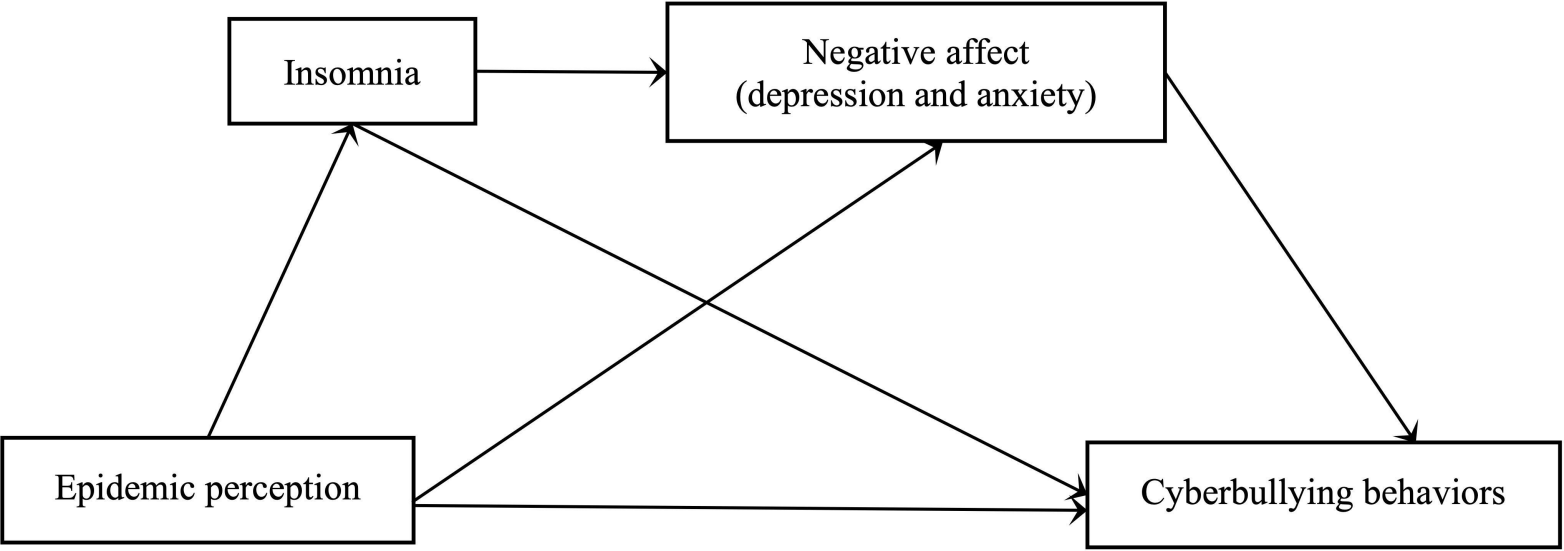
Serial mediating effect model diagram.

The aim of this study was to examine the relationship and mechanisms underlying the epidemic perception and cyberbullying behavior among adolescents. This study is the first to consider sleep problems and emotional problems as the chain mediating variables of the influence of psychological distress on cyberbullying. Although it has been suggested that sleep problems and negative affect are related to epidemic perception or cyberbullying behaviors, it remains unclear that how insomnia and negative affect influence this relationship. This is the first study to take both the mediating effects of insomnia and negative affect into consideration, which will contribute to a deeper understanding of the mechanisms by which the perception of COVID-19 pandemic and cyberbullying behaviors are connected, understanding and mitigating the profound impact of the COVID-19 pandemic in adolescents, and facilitate the development of GST hypothesis in the cyberbullying field. To conclude, this study tested the mediating effects of insomnia and negative affect on the epidemic perception-cyberbullying behaviors relationship using a sample of Chinese adolescents from a web-based survey in Shandong, China. Based on previous empirical research, we proposed three hypotheses: (1) epidemic perception exerts positive influences on cyberbullying behaviors; (2) insomnia mediates the relationship between epidemic perception and cyberbullying behaviors; (3) negative affect mediates the relationship between epidemic perception and cyberbullying behaviors; and (4) both insomnia and negative affect play a serial mediating effect on the relationship between epidemic perception and cyberbullying behaviors of adolescents. The theoretical mediating model is shown in Figure 1.

## Methods

### Participants

Data for this study were collected as part of a web-based survey. Based on the research’s necessity and feasibility, middle and high school students were identified as the target participants, with participants planned to be recruited from collaborating schools. We recruited 20,000 students from 35 junior and senior high schools in Shandong, China, with the cluster random sampling method in 2022. A total of 274 submitted questionnaires were discarded because of high levels of missing data or their answers were clearly fictitious or inconsistent. The final valid study population consisted of 19,726 students, including 10,371 boys (10,371/19,726, 52.58%), and the mean average age of the students was 14.80 years (SD 1.63). A total of 16,846 participants were non–only children (16,846/19,726, 85.40%). A total of 469 students were at grade 6 (469/19,726, 2.38%), 1892 were at grade 7 (1892/19,726, 9.59%), 11,630 were at grade 8 (11,630/19,726, 58.96%), 4361 were at grade 1 1 (4361/19,726, 22.11%), and 1373 were at grade 12 (1373/19,726, 6.96%).

### Ethical Considerations

In this study, informed consent was obtained from the head teacher, the primary guardian, and the participants themselves before distributing the questionnaires. The questionnaires were distributed to each class’s WeChat group via the Wenjuanxing platform. Participants completed the self-report questionnaires on the web, and the process took approximately 30 minutes. In addition, all procedures involving human participants in this study complied with the ethical standards of the Academic Board of Shandong Normal University and the 1964 Declaration of Helsinki and its later amendments.

Participation was entirely voluntary and anonymous, based on written informed consent, and participants had the right to withdraw from the study at any time.

To ensure participants’ privacy, all collected data were anonymized, and no personally identifiable information was collected or stored. All data were securely stored in a password-protected database, accessible only to authorized researchers. Participants’ responses were treated with strict confidentiality, and the research team took all necessary measures to safeguard their personal information.

Finally, to express our gratitude for the participants’ cooperation and support, each participant was compensated with 10 RMB. This compensation was intended as a reasonable acknowledgment of the time and effort they invested in completing the questionnaire and did not influence their willingness to participate.

This study was approved by the Academic Board of Shandong Normal University, in accordance with the ethical standards of the 1964 Declaration of Helsinki, under the approval number SDNU1003.

### Measures

#### Perception of Coronavirus Pneumonia Scale

To assess the change of behavior, cognition, and emotion of the public during COVID-19 pandemic, we used the Perception of Coronavirus Pneumonia Scale [39]. The self-report instrument included 5 items scored on a 5-point Likert scale of 1 (not at all true of me) to 5 (extremely true of me), including statements such as “I think anyone can get COVID-19.” The Cronbach α coefficient for this study is 0.80.

#### Mental Health Inventory of Middle School Students (MMHI-60)

To assess mental health, we used the Mental Health Inventory of Middle School Students (MMHI-60) [40]. This measure included 12 items measured on a 5-point Likert scale of 1 (never) to 5 (seriously). The MMHI-60 was developed for adolescents and is proved to be reliable and valid for application in China [41]. This study made use of 6 items pertaining to depression (eg, I felt depressed) and 6 items reflecting anxiety (eg, I felt restless and uneasy) to assess the levels of depression and anxiety among students. Higher scores indicate higher depression and anxiety. In this study, the Cronbach α coefficient for the whole scale is 0.90.

#### Sleep Problems Estimation Scale

Insomnia was measured by the Sleep Problems Estimation Scale developed by Jenkins et al [42] and revised by Zhang et al [43]. This 4-item questionnaire used a 5-point Likert scale of 1 (totally disagree) to 5 (totally agree) and included statements such as “I had difficulty falling asleep.” Higher scores indicated higher levels of insomnia. The Cronbach α coefficient in this study is 0.83.

#### Adolescent Online Aggressive Behavior Scale

Cyberbullying behaviors were evaluated with the Adolescent Online Aggressive Behavior Scale (AOABS) developed by Zhao and Gao [44]. AOABS consisted of 2 subscales, including instrumental aggression and reactive aggression. Each subscale was divided by 2 factors of overt aggression and relational aggression. The AOABS was developed for adolescents and is proved to be reliable and valid for application in China [45]. This measure included 15 items scored on a 4-point Likert scale of 1 (never) to 4 (always), including statements such as “I often insult others when playing online games.” Higher scores on this scale indicated more cyberbullying behaviors. The Cronbach α coefficient in this study is 0.80.

### Data Analysis

The collected 19,726 valid questionnaires were coded and entered into the database. Based on the data sets of all valid samples included in this study, we computed descriptive analyses and correlation between all variables. In this study, Pearson correlation analysis is used to analyze the correlation between variables, and chain mediation analysis is used to analyze the mediation role. In this study, the correlation of the 4 variables is analyzed first and then the chain mediation effect is further analyzed. SPSS (version 25.0; IBM Corp) and Mplus (version 7.0; Muthén) were used in this study.

## Results

### Preliminary Analyses

The collected data were tested for common method bias using Harman’s single-factor test. The results of the explanatory factor analysis extracted that the biggest factor accounting for 21.31% of the variance, which was far lower than 40% [46]. Although this process did not completely exclude the possibility of common method bias, the results showed that the data collected in this study did not lack severe common method bias. In addition, Mplus 7.0 statistical analysis software was used to perform confirmatory factor analysis on the observed data, and the results are shown in Table 1. From this, the results showed that the fitting degree between the observed data and the hypothesis model (4-factor model) was the best. RMSEA was 0.02, CFI was 0.98, TLI was 0.98, and SRMR was 0.04, which met the requirements. Based on the above results, it can be concluded that this study has a good discriminant validity among the tested variables.

Pearson correlation analysis was used to test the bivariate correlations of all the variables. As shown in Table 2, it was found that the epidemic perception of participants was positively correlated with insomnia (*r*=0.19; *P*<.001), anxiety and depression (*r*=0.25; *P*<.001), and cyberbullying behaviors (*r*=0.13; *P*<.001). And cyberbullying behaviors had a significantly positive correlation with insomnia (*r*=0.30; *P*<.001) and anxiety and depression (*r*=0.25; *P*<.001). Insomnia was positively corrected with anxiety and depression (*r*=0.44; *P*<.001) as well. For demographic variables, we found that age was positively associated with insomnia (*r*=0.01; *P*=.046) and cyberbullying behaviors (*r*=0.05; *P*<.001), and age was negatively associated with epidemic perception (*r*=−0.02; *P*=.001). Gender was negatively associated with epidemic perception (*r*=−0.03; *P*<.001) and cyberbullying behaviors (*r*=−0.11; *P*<.001), and gender was positively associated with anxiety and depression (*r*=0.02; *P*=.006). Age was negatively associated with gender (*r*=−0.25; *P*<.001). In addition, the gender and age of adolescents were significantly correlated with epidemic perception, anxiety and depression, and cyberbullying behaviors (Table 3). Thus, gender and age of adolescents were included as control variables in the subsequent analysis.

**Table 1.** Results of confirmatory factor analysis of the measurement models (n=19,726).

Model	Chi-square(*df*)		Chi-square/*df*	RMSEA^a^	CFI^b^	TLI^c^	SRMR^d^
Four-factor model (A^e^, B^f^, C^g^, D^h^)	5014.68(436)		11.50	0.02	0.98	0.98	0.04
Three-factor model (A, B+C, D)	177605.93(561)		316.59	0.10	0.53	0.47	0.13
Two-factor model (A+B + C, D)	232352.06(593)		391.82	0.12	0.38	0.34	0.14

a RMSEA: root mean square error of approximation.

b CFI: Comparative fit index.

c TLI: Tucker-Lewis index.

d SRMR: standardized root mean square residual.

e A: Epidemic perception.

f B: Insomnia.

g C: Anxiety and depression.

h D: Cyberbullying behaviors.

**Table 2.** Descriptive statistics and correlation analysis for epidemic perception, insomnia, negative affect, cyberbullying behaviors, and demographic variables (n=19,726).

	M(SD)	1	2	3	4	5	6
Epidemic perception	3.06(1.00)	N/A^a^					
Insomnia	1.61(0.72)	0.19(*P*<.001)	N/A				
Anxiety and depression	1.51(0.57)	0.25(*P*<.001)	0.44(*P*<.001)	N/A			
Cyberbullying behaviors	1.04(0.10)	0.13(*P*<.001)	0.30(*P*<.001)	0.25(*P*<.001)	N/A		
Age	14.80(1.63)	−0.02(*P*=.001)	0.01(*P*=.046)	0.01	0.05(*P*<.001)	N/A	
Gender	1.47(0.50)	−0.03(*P*<.001)	0.01	0.02(*P*=.006)	−0.11(*P*<.001)	−0.25(*P*<.001)	N/A

a Not applicable.

**Table 3.** Results of serial mediating effect for insomnia and negative affect (n=19,726).

Mediating path	Effect value	Boot SE	95% CI
Total mediating effect	0.079	0.003	0.074-0.084
Epidemic perception→insomnia→cyberbullying	0.044	0.002	0.040-0.048
Epidemic perception→depression and anxiety→cyberbullying	0.024	0.002	0.021-0.027
Epidemic perception→insomnia→depression and anxiety→cyberbullying	0.011	0.001	0.010-0.013

### Test for Structural Model

The structural equation model was used to investigate the mediating effect of insomnia and depression and anxiety. The maximum likelihood estimation method is used to estimate the mediation effect. The hypothesized structural model included epidemic perception as exogenous variable and insomnia, anxiety and depression, and cyberbullying behaviors as endogenous variables. As shown in Figure 1, epidemic perception (*β*=.04; *P*<.001), insomnia (*β*=.24; *P*<.001), and anxiety and depression (*β*=.14; *P*<.001) positively predicted cyberbullying behaviors; insomnia (*β*=.44; *P*<.001) and epidemic perception (*β*=.14; *P*<.001) positively predicted anxiety and depression; and epidemic perception (*β*=.15; *P*<.001) positively predicted insomnia.

**Figure 2.**
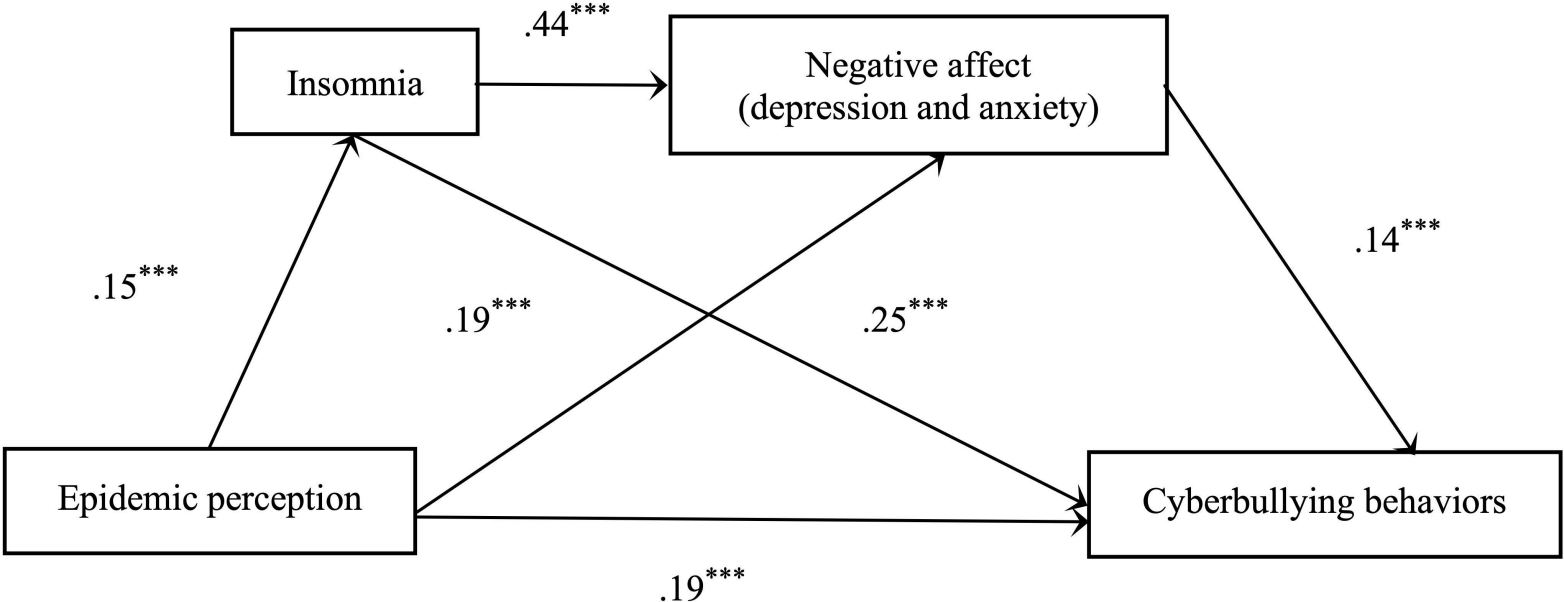
The serial mediating effect model of insomnia and negative affect. (**P<.01, ***P<.001).

The results of bootstrap revealed that epidemic perception had significant direct effect on cyberbullying behaviors (direct effect=0.040, SE=0.005; *P*<.001; 95% bootstrap CI 0.050-0.060) and significant indirect effects on cyberbullying behaviors via insomnia (indirect effect=0.040, SE=0.002; *P*=.01; 95% bootstrap CI 0.032-0.040). Epidemic perception had significant indirect effects on cyberbullying behaviors via anxiety and depression (indirect effect=0.020, SE=0.001; *P*=.01; 95% bootstrap CI 0.016-0.022). The epidemic perception had significant indirect effects on cyberbullying behaviors via insomnia and anxiety and depression (indirect effect=0.009, SE =0.001; *P*=.01; 95% bootstrap CI 0.007-0.010). These results indicated that insomnia and negative emotions (anxiety and depression) mediated the relation between epidemic perception and cyberbullying behaviors, which are shown in Figure 2.

## Discussion

### Principal Findings

In this study, we investigated the effect of epidemic perception on cyberbullying behaviors of Chinese adolescents with a serial mediating model. The results showed that epidemic perception is positively correlated with cyberbullying behaviors. Insomnia and negative affect play independent mediating and serial mediating roles in epidemic perception and cyberbullying behaviors.

Relevant research shows that adolescent cyberbullying has increased significantly during the epidemic [47]. The environment-related stressors, which means stressors not depending on an individual’s behavior, are correlated with bullying [48]. Although it could be especially true during the COVID-19 pandemic, no research has explored the mechanism. Given that, this study set out to explore the potential impact of COVID-19 perception on cyberbullying behavior among adolescents, revealing the potential mediating roles of insomnia and negative affect. Theoretically, this study contributes to the GST theory in important ways, which draws a useful comprehensive theoretical framework indicating that adolescents under stress are likely to experience a negative affective state, which mediates the relationship between stressors and aggressive behaviors. By conceptualizing the COVID-19 pandemic as a special situation factor that contributes to aggression behaviors, this study delineates how epidemic perception influences cyberbullying behaviors through insomnia and negative affect. The current findings emphasized a supportive model for how cyberbullying behaviors develop during the crisis, which provide important directions of interventions for adolescents to consider in dealing with the COVID-19 crisis.

The direct pathway from epidemic perception to cyberbullying behaviors of adolescents was significant, implying that the perception of COVID-19 pandemic could directly influence cyberbullying behaviors over time. Furthermore, understanding factors that increase risk for cyberbullying behaviors is a critical goal for research, and this study focuses on sleep problems and negative affect as potential risk factors, and it is worth noting that the full mediating effect model corroborated the GST theory. At the input state, situational factors influence aggressive behaviors by acting on the individuals’ internal states (arousal, emotions, and other internal states), which means adolescents who experience high levels of epidemic perception are more likely to experience insomnia and be anxious or depressive, which become risk factors for cyberbullying. Therefore, it is possible that treating the internal states (ie, insomnia or negative affect) may mitigate some of the effects of epidemic perception on adolescent cyberbullying.

Turning first to the main question of mediating effects, our findings converge with the previous hypothesis. The path analysis results demonstrated that the perception of epidemic indirectly influenced cyberbullying via 3 pathways: insomnia, negative affect, and the serial mediating effect of insomnia, depression, and anxiety. First, based on the current findings, insomnia is suggested as a strong linkage between epidemic perception and cyberbullying behaviors. Theoretical models suggest that insomnia symptoms can arise due to acute stressors, changes in routines, or changes in life circumstances, all of which are categorized as precipitating factors. Specifically, in the developmentally sensitive times, adolescents may bear more stress due to the perception and worry of epidemic, experiencing disturbed sleep or nightmares. It is possible that the worry is a risk factor of the relationships between COVID-19 stressors and insomnia [49]. The restrictions and feeling of worry surrounding the social confinement have upset daily routines that typically serve as timekeepers for sleep-wake rhythms, which might explain the increased incidence of insomnia. Consistent with studies available, this study found that adolescents try to engage in aggression to get crucial behavioral and mental functions needed in experiencing insomnia. Sleep disturbances have recently been explored as a potential factor that may contribute to, or be a consequence of, involvement in bullying [27]. From the perspective of neurophysiological, related with amygdala reactivity and the connectivity between the amygdala and medial prefrontal cortex [44], sleep deprivation appears to intensify emotional experience, while at the same time reducing the ability to control those emotions, potentially leading to aggression. During COVID-19 pandemic, when confined at home, adolescents spend more time on social media and shift focus from offline activities to online activities, so they are more likely to externalize these aggressive behaviors into cyberbullying.

In addition, we also confirmed the mediating effect of negative emotions, that is, adolescents with higher level of epidemic perception are prone to negative emotions, which in turn lead to more cyberbullying behaviors. To be specific, as an acute stressor, the COVID-19 pandemic has a significant effect on adolescents’ mental health, which has been documented of an increasing body of literature [36]. Exposure to stress may cause changes in physiology, including immune and cardiovascular system, which in turn harms one’s mental well-being [49]. Adolescents chronically exposed to COVID-19 information are more possible to experience distress, anxiety, and depression [48,49], which could be seen as frustration under pressure in the frustration-aggression model. The frustration increased adolescents’ feelings of aggressive inclinations. They relieve their additive anxiety and depression by being active on the web, thus developing maladaptive web-based behaviors such as cyberbullying.

Furthermore, we found that insomnia and negative emotions played a serial mediating role in the relationship between epidemic perception and cyberbullying. This literature suggests that aggression and behavior problems may result from insufficient or poor-quality sleep via 3 pathways: affect (eg, anger and irritability); behavior (eg, reduced ability to inhibit negative impulses, aggressive behaviors); and cognition (eg, negative evaluation of others contributes to risk of hostile or aggressive responses) [23]. In the serial mediating model, we found that there is a strong correlation between insomnia and negative affect, which suggests that under the perception of epidemic, adolescents’ emotion and sleep problems are inseparable. Insomnia has many negative psychological and emotional effects on adolescents such as verbal aggression, depression, and anxiety [50]. These additive feelings are direct and powerful driving forces, which prompt them to take cyberbullying behaviors. Our results give evidence of multifinality where cyberbullying behaviors during COVID-19 pandemic were associated with multiple factors, which provide comprehensive support for the significance of fear of epidemic perception in predicting cyberbullying behaviors. Furthermore, China has the tradition of showing great respect to teachers, and observing family piety rigidly [43] which represents a conformity orientation reflecting love and discipline, often at the cost of children’s autonomy and independence, making their perceived sense of self-awareness and competence be easily diminished [51]. When faced with stresses and web-based learning environments, Chinese children are at high risk to perpetrate cyberbullying as a way to alleviate negative feelings. For example, a latest longitudinal study conducted among Chinese adolescents found that prevalence rates of cyberbullying were 35.4% in November 2016, 25.2% in May 2017, and 23% in November 2017 [52].

### Limitations

Several limitations should be considered in this study to warrant further research. First, our data were cross-sectional, so we were unable to make a causal or temporal inference. Longitudinal associations between the epidemic perception, depression, anxiety, sleeping problems, and cyberbullying should be examined in future studies. Second, all participants were recruited from the mainland of China. Therefore, future research should explore whether the results of this study can be generalized to different cultural background groups. Also, future research should directly explore the effect of Chinese cultural factors on cyberbullying behaviors of Chinese adolescents and ultimately determine the specific direction for effective intervention in the future. Third, although common method bias in this study was not statistically significant, the social expectations in the measurements could not be ignored as all the data were obtained through adolescent self-report. We recommend that future studies can collect data from different sources. What is more, only individual variables affecting the relationship between epidemic perception and adolescent cyberbullying were examined in this study. Future research should examine interpersonal or environmental variables to further explore the causes of cyberbullying as well as the relationship between epidemic perception and cyberbullying.

### Practical Implications

Despite these limitations, our findings have significant implications for the prevention and intervention of adolescent cyberbullying behaviors. Findings from this study are consistent with a large amount of preexisting literature demonstrating that the outbreak of COVID-19 pandemic has long-term indirect psychopathological consequences to adolescents [48] including being a risk factor for cyberbullying behaviors. Reducing distress caused by the perception of COVID-19 pandemic, therefore, is likely to reduce cyberbullying behaviors of adolescents. Also, as the present findings suggested that inadequate sleep during the epidemic might lead to a possible predisposition in adolescents for affective and behavioral disinhibition, which further increases later cyberbullying, thus, improving the quality of sleep should be the focus of interventions that aimed at decreasing the risk of adolescent cyberbullying. In addition, given that negative affect also mediates the relation between epidemic perception and adolescent cyberbullying behaviors, emotion regulation self-help intervention may help reduce adolescent cyberbullying. The self-help emotion regulation strategies such as mindfulness and self-talk could help adolescents learn how to self-manage and ease individual anxiety and depression during the epidemic and become more peaceful, which could further reduce aggression feelings and cyberbullying [53]. At last, because this study showed that there are multiple mediators linking epidemic perception to adolescent cyberbullying behaviors, interventions that target the 2 mediators simultaneously are more likely to be effective than interventions that target any singular mediator.

### Conclusions

During the COVID-19 pandemic in China, the lockdown and epidemic perception significantly impacted the daily rhythm and structure of adolescents. Our study provided evidences that adolescents with high levels of epidemic perception are at great risk of cyberbullying, and insomnia, depression, and anxiety play mediating roles in this relationship. Theoretically, it broadened the way in which we understand adolescents’ psychological and behavioral characteristics and explore the behavior mechanism of cyberbullying during the COVID-19 outbreak. Practically, this research emphasized the need for social concern of mental health among adolescents. Society and parents ought to pay attention to their children’s sleep problems and levels of depression and anxiety to reduce cyberbullying behaviors during the COVID-19 outbreak.

## Abbreviations

AOABS: Adolescent Online Aggressive Behavior Scale
GST: General Strain Theory
MMHI-60: Mental Health Inventory of Middle School Students

## References

[1] Debnath B, Singh WS, Manna K (2021). The outbreak of coronavirus disease 2019 (COVID-19) and its manifestation. COVID.

[2] Brooks SK, Webster RK, Smith LE (2020). The psychological impact of quarantine and how to reduce it: rapid review of the evidence. Lancet.

[3] Ferry F, Bunting B, Rosato M, Curran E, Leavey G (2021). The impact of reduced working on mental health in the early months of the COVID-19 pandemic: results from the understanding society COVID-19 study. J Affect Disord.

[4] Shen K, Yang Y, Wang T (2020). Diagnosis, treatment, and prevention of 2019 novel coronavirus infection in children: experts’ consensus statement. World J Pediatr.

[5] Becker SP, Gregory AM (2020). Editorial perspective: perils and promise for child and adolescent sleep and associated psychopathology during the COVID‐19 pandemic. Child Psych Psychiatry.

[6] Jefsen OH, Rohde C, Nørremark B, Østergaard SD (2021). Editorial perspective: COVID‐19 pandemic‐related psychopathology in children and adolescents with mental illness. Child Psych Psychiatry.

[7] Pedrini L, Meloni S, Lanfredi M (2022). Adolescents’ mental health and maladaptive behaviors before the COVID-19 pandemic and 1-year after: analysis of trajectories over time and associated factors. Child Adolesc Psychiatry Ment Health.

[8] Marinucci M, Pancani L, Aureli N, Riva P (2022). Online social connections as surrogates of face-to-face interactions: a longitudinal study under COVID-19 isolation. Comput Hum Behav.

[9] Livazović G, Ham E (2019). Cyberbullying and emotional distress in adolescents: the importance of family, peers and school. Heliyon.

[10] Smith PK, Mahdavi J, Carvalho M, Fisher S, Russell S, Tippett N (2008). Cyberbullying: its nature and impact in secondary school pupils. J Child Psychol Psychiatry.

[11] Agnew R (1992). Foundation for a general strain theory of crime and delinquency. Criminology.

[12] López-Vázquez E, Marvan ML (2003). Risk perception, stress and coping strategies in two catastrophe risk situations. Soc Behav Pers.

[13] Han Q, Zheng B, Agostini M (2021). Associations of risk perception of COVID-19 with emotion and mental health during the pandemic. J Affect Disord.

[14] Slovic P (1987). Perception of risk. Science.

[15] Siegrist M, Keller C, Kiers HAL (2005). A new look at the psychometric paradigm of perception of hazards. Risk Anal.

[16] Loewenstein GF, Weber EU, Hsee CK, Welch N (2001). Risk as feelings. Psychol Bull.

[17] Bailen NH, Green LM, Thompson RJ (2019). Understanding emotion in adolescents: a review of emotional frequency, intensity, instability, and clarity. Emotion Rev.

[18] Olino TM, Lopez-Duran NL, Kovacs M, George CJ, Gentzler AL, Shaw DS (2011). Developmental trajectories of positive and negative affect in children at high and low familial risk for depressive disorder. J Child Psychol Psychiatry.

[19] Zhang Y, Xu C, Dai H, Jia X (2021). Psychological distress and adolescents’ cyberbullying under floods and the COVID-19 pandemic: parent–child relationships and negotiable fate as moderators. Int J Environ Res Public Health.

[20] Dahl RE (2006). Sleeplessness and aggression in youth. J Adolesc Health.

[21] Ireland JL, Culpin V (2006). The relationship between sleeping problems and aggression, anger, and impulsivity in a population of juvenile and young offenders. J Adolesc Health.

[22] Gregory AM, Sadeh A (2012). Sleep, emotional and behavioral difficulties in children and adolescents. Sleep Med Rev.

[23] Krizan Z, Herlache AD (2016). Sleep disruption and aggression: implications for violence and its prevention. Psychol Violence.

[24] Killgore WDS (2013). Self-reported sleep correlates with prefrontal-amygdala functional connectivity and emotional functioning. Sleep.

[25] Yoo SS, Gujar N, Hu P, Jolesz FA, Walker MP (2007). The human emotional brain without sleep--a prefrontal amygdala disconnect. Curr Biol.

[26] Lippi G, Henry BM, Sanchis-Gomar F (2021). Putative impact of the COVID-19 pandemic on anxiety, depression, insomnia and stress. Eur J Psychiatry.

[27] Chen F, Zheng D, Liu J, Gong Y, Guan Z, Lou D (2020). Depression and anxiety among adolescents during COVID-19: a cross-sectional study. Brain Behav Immun.

[28] Jiao WY, Wang LN, Liu J (2020). Behavioral and emotional disorders in children during the COVID-19 epidemic. J Pediatr.

[29] Fahy AE, Stansfeld SA, Smuk M, Smith NR, Cummins S, Clark C (2016). Longitudinal associations between cyberbullying involvement and adolescent mental health. J Adolesc Health.

[30] Kowalski RM, Limber SP (2013). Psychological, physical, and academic correlates of cyberbullying and traditional bullying. J Adolesc Health.

[31] Tesser A, Leone C (1977). Cognitive schemas and thought as determinants of attitude change. J Exp Soc Psychol.

[32] Calvete E (2014). Emotional abuse as a predictor of early maladaptive schemas in adolescents: contributions to the development of depressive and social anxiety symptoms. Child Abuse Negl.

[33] Llorca A, Malonda E, Samper P (2016). The role of emotions in depression and aggression. Med Oral Patol Oral Cir Bucal.

[34] Spithoven AWM, Lodder GMA, Goossens L (2017). Adolescents’ loneliness and depression associated with friendship experiences and well-being: a person-centered approach. J Youth Adolesc.

[35] Bajaj S, Blair KS, Schwartz A, Dobbertin M, Blair RJR (2020). Worry and insomnia as risk factors for depression during initial stages of COVID-19 pandemic in India. PLoS One.

[36] Zhang C, Ye M, Fu Y (2020). The psychological impact of the COVID-19 pandemic on teenagers in china. J Adolesc Health.

[37] Baglioni C, Battagliese G, Feige B (2011). Insomnia as a predictor of depression: a meta-analytic evaluation of longitudinal epidemiological studies. J Affect Disord.

[38] Blanken TF, Borsboom D, Penninx BW, Van Someren EJ (2020). Network outcome analysis identifies difficulty initiating sleep as a primary target for prevention of depression: a 6-year prospective study. Sleep.

[39] Qian MY, Ye DM, Behavior DW (2003). Cognition and emotion of the public in Beijing towards SARS. Chin Ment Health J.

[40] Wang JS, Li Y, He ES (1997). Development and standardization of mental health inventory of middle-school students in China. Sci of Soc Psychol.

[41] Ren ZH, Jiang GR (2011). Development of school mental health education satisfaction evaluation scale. Chin Ment Health J.

[42] Jenkins CD, Stanton BA, Niemcryk SJ, Rose RM (1988). A scale for the estimation of sleep problems in clinical research. J Clin Epidemiol.

[43] Zhang X, Zhao C, Niu Z, Xu S, Wang D (2021). Job insecurity and safety behaviour: the mediating role of insomnia and work engagement. Int J Environ Res Public Health.

[44] Zhao F, Gao WB (2012). Evaluation of internet aggression behavior scale for adolescent. Chin J Ment Health.

[45] Wang JS, Li Y, He ES (1997). Development and standardization of mental health inventory of middle-school students in China. Sci Soc Psychol.

[46] Zhou H, Long LR (2004). Statistical remedies for common method biases. Adv Psychol Sci.

[47] Yang F (2021). Coping strategies, cyberbullying behaviors, and depression among Chinese netizens during the COVID-19 pandemic: a web-based nationwide survey. J Affect Disord.

[48] Park S, Dotterer AM (2018). Longitudinal associations of family stressors, fathers’ warmth, and Korean children’s externalizing behaviors. J Fam Psychol.

[49] Bøe T, Serlachius AS, Sivertsen B, Petrie KJ, Hysing M (2018). Cumulative effects of negative life events and family stress on children’s mental health: the Bergen Child study. Soc Psychiatry Psychiatr Epidemiol.

[50] Alvaro PK, Roberts RM, Harris JK (2014). The independent relationships between insomnia, depression, subtypes of anxiety, and chronotype during adolescence. Sleep Med.

[51] Givertz M, Segrin C (2012). The association between overinvolved parenting and young adults’ self-efficacy, psychological entitlement, and family communication. Comm Res.

[52] Chu XW, Fan CY, Liu QQ, Zhou ZK (2018). Stability and change of bullying roles in the traditional and virtual contexts: a three-wave longitudinal study in Chinese early adolescents. J Youth Adolesc.

[53] Schonfeld IS, Chang CH, Wang D, Hu YT (2022). Occupational Health Psychology.

